# Thymol, as a Succedaneum for Arsenic, in Pulpitis

**Published:** 1899-02

**Authors:** 


### Thymol, as a Succedaneum for Arsenic, in Pulpitis.

Thymol is said to have been used since the past several months, with very satisfactory results. This may be of interest to physicians practicing in the country or on board ship. A plug of cotton charged with a few fine particles of thymol is simply inserted into the cavity of the aching tooth; if the offending tooth cannot be readily located (as is very often the case in pulpitis), thymol should be applied to all suspicious teeth on the painful side. This plan may be carried out in children without danger, considering the small and harmless amount of thymol used. Should any of the thymol reach the tongue or the mucous membrane of the mouth, rinsing will counteract ill effects which might follow. Thymol is, in a degree, benumbing to the tongue and mucous membrane, but not on the uninjured external skin. Coarse particles dissolve very slowly in the cavity of the tooth. If a more rapid solution and more energetic action is desired, the patient should cleanse the mouth repeatedly with warm water. Thymol has never—as occurs so frequently with arsenic—increased the pain at the moment of its application. The insertion of thymol must be repeated several days in most cases, to prevent recurrence of pain. If the diseased tooth is to be preserved by filling, dental advice must be sought.—*Wiener Med. Press*, 1893, *No.* 37.

A Very Sad Dental Mishap is reported from Adelaide, South Australia. In June last a young lady named Maud Catchlove had two teeth extracted by Mr. B. Thomson, dentist, of King William Street. The operation was followed by coughing and symptoms of chest irritation, and subsequently abscess developed on the lung. Medical attendance was secured, but the patient gradually became worse, and died on July 28th. A post-mortem examination revealed a tooth in the first division of the left bronchus, and at the close of the inquiry the jury found that death was caused by the tooth found in the bronchus, and added a rider to the effect that Mr. Thomson should be more careful in extracting teeth. Evidence was given at the inquest that after the dental operation Mr. Thomson requested the friend of the patient who accompanied her to leave the room, and that he then inverted the patient and tapped her on the back. Mr. Thomson, however, denied that he had any suspicion that a tooth had gone down, and in explanation of the inversion said that he feared some pus from the abscess at the root of the tooth had gone down into the lung, and did this to get rid of it. Had information been given at once as to the probability of a tooth having been swallowed, an operation might, according to the medical evidence, have saved the patient's life.—*Pharmaceutical Journal.*

A mixture of equal parts of lactic acid and glycerine, applied to the face twice a day as a wash, is of value in removing freckles.—*Med. Brief.*

